# Automated ICD-10–Anchored Classification of Primary Care Text Data: Development and Evaluation of a Custom Multilabel Classifier

**DOI:** 10.2196/86533

**Published:** 2026-04-06

**Authors:** Christina Haag, Thomas Grischott, Jakob M Burgstaller, Stefan Markun, Oliver Senn, Viktor von Wyl

**Affiliations:** 1 Epidemiology, Biostatistics and Prevention Institute, University of Zurich, Hirschengraben 84, Zurich, 8001, Switzerland, +41 44 63 46380; 2 Institute for Implementation Science in Health Care, University of Zurich, Universitätstrasse 84, Zurich, 8006, Switzerland, 41 44 63 46380; 3 Institute of Primary Care, University Hospital Zurich, University of Zurich, Zurich, Switzerland

**Keywords:** electronic medical records, free-text classification, ICD-10 coding, language models, natural language processing, automated coding, primary care, clinical text mining, health informatics

## Abstract

**Background:**

Electronic medical records are a vast and valuable source of information, useful for tasks such as estimating disease prevalence. However, in routine primary care, much of this information is in free-text format rather than in a structured form and, therefore, not readily amenable to analysis. Manual coding of this textual data is both time-consuming and resource-intensive, making it impractical for large datasets. Although powerful open-source language models offer new opportunities for automated coding, their use on short heterogeneous primary care notes, particularly in German-language settings, remains insufficiently studied.

**Objective:**

By providing hands-on guidance for applied health researchers, this study aims to demonstrate the effective and accurate automatic classification of free-text notes using a language model fine-tuned for automated *International Statistical Classification of Diseases, Tenth Revision* (*ICD-10*) coding.

**Methods:**

Building on the extensive Family Medicine Research Using Electronic Medical Records (FIRE) routine database from the Institute of Primary Care at the University Hospital Zurich and the University of Zurich, we trained a large language model–based multilabel classifier on a dataset of 38,728 free-text notes, which had been manually categorized into 47 classes using specific *ICD-10* codes and code ranges or nondiagnostic/ad hoc labels (eg, “unclear diagnosis,” “status post”). We stratified the labeled data into training (70%), validation (15%), and posttraining test (15%) sets, ensuring similar label distributions across these sets. Using the Transformers Python library, we trained the model over 10 epochs and evaluated it on the posttraining test dataset.

**Results:**

Across 48 classes, the FIRE classifier achieved strong performance on the held-out posttraining set, with *F*_1_-scores of 0.85 (micro, overall across all predictions), 0.86 (macro, mean of per-class scores treating classes equally), and 0.84 (weighted, per-class scores weighted by class frequency).

**Conclusions:**

This study demonstrates steps for training open-source large language models and highlights the potential to streamline and scale the extraction of diagnostic information for practical applications. Our model can be robustly deployed, for example, for prescreening and labeling of free-text information, thus potentially reducing the burden of repetitive and error-prone manual handling.

## Introduction

Electronic medical records (EMRs) contain rich diagnostic information, but in routine primary care, much of this information is documented in short unstructured free-text format rather than as structured records. As a result, large-scale analysis and reuse of diagnostic data remains challenging, since manual coding of free-text notes is both time-consuming and resource-intensive, making it impractical on a large scale. Therefore, automated assignment of diagnostic codes, in particular from the *International Statistical Classification of Diseases, Tenth Revision* (*ICD-10*), has long been of practical interest for research and health care.

The emergence of powerful open-source language models offers new opportunities to automate text-based diagnosis extraction for research or health care. Transformer-based models like bidirectional encoder representations from transformers (BERT) and generative pretrained transformers have gained traction for *ICD-10* coding of clinical texts, achieving strong performance on English language EMR datasets [1,2]. However, research on the classification of text data into *ICD-10*–anchored classes has been conducted mainly in English [1]. While recent studies demonstrate that domain-adapted transformer models can perform well on non-English clinical texts—including Portuguese [3], French [4], and Spanish [5,6]—the availability of data and pretrained models varies widely across languages. For German clinical texts, resources remain limited, and existing work has largely focused on well-structured, high-quality documents such as discharge summaries or surgery reports. In routine primary care documentation in particular, notes are often short, loosely structured, and heterogeneous. Diagnostic coding may also combine *ICD-10* codes with administrative or primary care–specific systems such as the *International Classification of Primary Care*. These characteristics limit the direct reuse of existing models and underscore the need for approaches that are robust to real-world data and adaptable to local coding practices.

Taken together, these factors—a scarcity of annotated German clinical text datasets from routine care, the predominance of English language research, and the short heterogeneous nature of primary care notes—leave little guidance on how to adapt open-source large language models (LLMs) for routine use in German primary care. To address this gap, our study describes the implementation of a flexible, German-language, multilabel LLM classifier for real-world free-text notes in primary care. Although focused on a German-language dataset, the methodological approach may serve as a blueprint in settings beyond English.

## Methods

### Data Source: The Family Medicine Research Using Electronic Medical Records Database

The Swiss Family Medicine Research Using Electronic Medical Records (FIRE) database was initiated in 2009 and has been designed as a basic knowledge base for primary care research through the use of EMRs [7]. So far, the FIRE project has enrolled around 450 general practitioners throughout Switzerland who are willing to contribute their patients’ EMRs to the database. General practitioners in the FIRE project routinely document patient encounters—in clinics, at home, or by phone—and records are regularly exported to the FIRE database. The reasons for physician encounters are encoded using the *International Classification of Primary Care, 2nd edition*, albeit not systematically by all participating clinicians. Instead, many participating physicians often include unstructured free-text notes, particularly in the health problems and diagnoses fields, where diagnostic and health care use information is recorded.

### Ethical Considerations

The local ethics committee of the Canton of Zurich waived approval for research with the FIRE database because patient data is fully anonymized and, therefore, outside the scope of the Swiss Human Research Act (BASEC-Nr. Req2017– 00797). The study was conducted in accordance with the Declaration of Helsinki and Good Clinical Practice guidelines.

### Diagnosis and Problem List Free-Text Notes

This study used manually annotated free-text notes from the diagnosis and problems lists in Swiss general practitioners’ EMRs. In Swiss primary care, these lists function as longitudinal note-based records with brief entries summarizing patients’ current and past health problems (eg, “hypertension,” “low back pain”) as well as related events or observations. Each note represents a single, concise observation that persists across consultations, as structured diagnostic coding is not routinely used in Swiss outpatient care.

### Training an LLM-Based Classifier for Automated Text Classification

#### Overview

**Figure 1.**
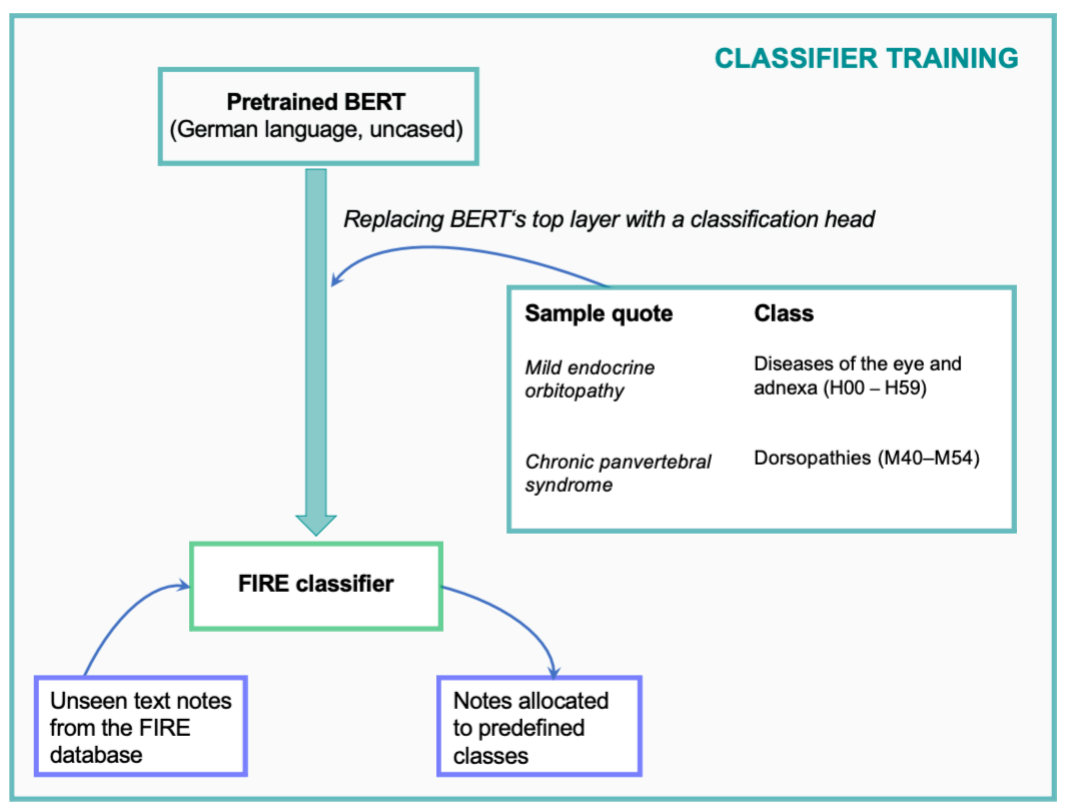
The figure visualizes the modeling procedure to build the multilabel classifier based on the FIRE dataset. BERT: bidirectional encoder representations from transformers; FIRE: Family Medicine Research Using Electronic Medical Records.

All analyses and visualizations were performed in Python (version 3.11; Python Software Foundation), using the Jupyter Notebook environment (version 2024.3.2; Project Jupyter). Individual analysis steps and specific Python libraries are detailed in the following sections. The overall procedure is visualized in Figure 1. The Jupyter Notebook containing the Python code and complete model training output is provided in Multimedia Appendix 1.

#### Step 1: Ground Truth Data for Classifier Training

We worked with a total of 53,481 free-text notes, incorporating data from Wallnöfer et al [8] and additional notes annotated thereafter (n=26,501, 49.6%). Wallnöfer et al [8] developed an *ICD-10*–anchored framework comprising 105 diagnostic codes, including both individual *ICD-10* codes and code ranges, as well as a nonspecific “no diagnosis” category. For the present study, a subset of these codes was consolidated into broader groups by combining related diagnostic categories into higher-level ICD classifications, resulting in 44 categories. In addition, three less specific encounter codes not included in the original framework were introduced—“suspected diagnosis,” “unclear,” and “status post”—bringing the total to 47 categories. Reported interrater reliability for the original codes used in this study—either directly or in aggregated form—ranged from moderate to almost perfect (κ=0.53-0.98); however, these values are not directly transferable to the aggregated categories. For the Wallnöfer et al [8] dataset (n=26,980, 50.4%), two physician raters independently coded the notes (full details are reported in Wallnöfer et al [8]). The remaining 26,501 (49.6%) notes were annotated by a single trained physician.

#### Step 2: Data Preprocessing

To create a more balanced dataset, we reduced the number of entries for the most heavily overrepresented *ICD-10* code range Z00-Z99 “factors influencing health status and contact with health services,” which occurred in 42.5% (n=22,714) of all free-text notes, either alone or in combination with other labels. Specifically, we removed all 14,316 free-text entries assigned exclusively to this code range, reducing the overall dataset to 38,728 free-text notes. As a result, this code range’s representation was reduced to 8226 (21.2%), reflecting only those entries where Z00-Z99 appeared alongside other labels.

#### Step 3: Subdividing the Data Into Training, Validation, and Posttraining Test Data

We then divided the preprocessed dataset (n=38,728) into three parts: 70% (n=27,108) for training, 15% (n=5810) for validation during training, and 15% (n=5810) for final testing. To ensure each class label was proportionally represented in all three sets, we used a stratified splitting process based on the final list of 47 labels, using the *iterstrat* Python package and its MultilabelStratifiedShuffleSplit class for multilabel stratified splitting. In other words, if a particular class made up, for instance, 10% of the entire dataset, it also contained roughly 10% of the training set, 10% of the validation set, and 10% of the posttraining test set.

#### Step 4: Model Training

To build our classifier, we used a German BERT LLM (dbmdz/bert-base-german-uncased) from the Hugging Face model hub [9] and replaced its top layer with a classification head to identify the 47 classes in unstructured medical free text. Tokenization was performed using the pretrained tokenizer associated with GermanBERT, a German-language–specific model; no multilingual or custom tokenization was applied (see executed Python script in Multimedia Appendix 1). The model’s maximum input length was 512 tokens. No truncation was applied during training or inference, and the model always processed the complete note (median 15 tokens, mean 18 tokens, maximum 111 tokens). Since a single free-text note may belong to multiple categories, we used a multilabel classification approach to allow the assignment of several labels to each note. For model training, we used the Trainer class from the Transformers [10] Python library, which streamlines model training and metric calculation. We set the decision threshold for the binary predictions (true/false) at 0.5 per class.

#### Step 5: Classifier Evaluation During Training

We monitored evaluation metrics across all 10 epochs throughout training. During each epoch, the model iterated over all training batches, updating its weights via backpropagation based on the binary cross-entropy training loss.

##### Overall Model Evaluation

After each epoch, we evaluated model quality on the validation set. We first recorded training loss—the average discrepancy between predictions and ground truth on the training data; lower is better. We computed the same metric on the validation set (validation loss) to assess generalization: if validation loss falls and then rises while training loss keeps falling, the model is starting to memorize rather than generalize. We also tracked the microaveraged *F*_1_-score (the harmonic mean of precision and recall), which gives more weight to more frequent classes. In addition, we measured the area under the receiver operating characteristic curve, summarizing how well the model separates positives from negatives across all thresholds (values near 1 indicate excellent separation; values near 0.5 indicate chance performance). Finally, because examples can have multiple labels, we reported subset (exact match) accuracy on the validation set—the fraction of examples for which every label is predicted correctly.

##### Per-Class Evaluation

Because each free-text note could belong to multiple classes, we examined model performance for each label individually using precision, recall, and *F*_1_-score metrics. These metrics are particularly informative for multilabel classification because they capture how well the model identifies positive cases (recall), avoids false positives (precision), and balances both aspects (*F*_1_-score). Accuracy was not used on a per-class basis, as it is dominated by true negatives in imbalanced datasets and may, therefore, provide a misleading indication of classifier performance.

### Step 6: Classifier Evaluation on the Posttraining Test Data

The classifier was subsequently evaluated on the held-out 15% posttraining test set, which had not been used during model training or validation.

#### Overall Model Evaluation

To provide an overall measure of performance across all diagnostic labels, we calculated the micro-, macro-, and weighted averages of precision, recall, and *F*_1_-score. The microaveraged metrics combine all true and false positives and negatives across labels, capturing the model’s overall ability to classify instances correctly. The macroaveraged scores assign equal weight to each label, offering a balanced view that is not influenced by class frequency. In contrast, the weighted average metrics account for label prevalence, summarizing performance in proportion to how often each label occurs in the data.

#### Per-Class Evaluation

To complement the aggregate measures, the classifier’s performance was also examined for each diagnostic category in the posttraining test set. For every label, precision, recall, and *F*_1_-score were computed based on the corresponding ground truth and predicted binary vectors. These per-class metrics highlight how accurately the model distinguishes between individual diagnostic groups, allowing detailed insight into which classes were predicted most reliably and which proved more challenging.

## Results

### Overview

On average, each free-text note was assigned to 1.27 different classes, with the number of classes per free-text note ranging from 1 to 4. The class with the highest number of free-text notes was the umbrella class “factors influencing health status and contact with health services” (Z00-Z99; n=8226). Other classes with particularly high counts included “status post diagnosis” (n=3655) and “diseases of the musculoskeletal system and connective tissue” (M00-M99; n=2883). Conversely, classes such as “dementia” (F00-F03; n=97), “heart failure” (I50; n=116), and “acute upper respiratory infections” (J00-J06; n=163) were among those with the lowest number of free-text notes.

### Monitoring Model Metrics

All model metrics during training and for the test data are provided in Multimedia Appendix 1.

### Model Metrics During Training

Over the course of 10 training epochs, overall model performance reached a plateau, with the highest *F*_1_-score reaching 0.85 achieved at epoch 10, indicating convergence of model learning. Overall model accuracy at this point was 0.75, and the area under the receiver operating characteristic curve equaled 0.92. The trained FIRE classifier demonstrated robust performance across the majority of classes; specifically, precision during training averaged 0.88 (SD 0.08), recall averaged 0.84 (SD 0.14), and the *F*_1_-score averaged 0.86 (SD 0.11).

### Performance on Posttraining Test Data

**Figure 2.**
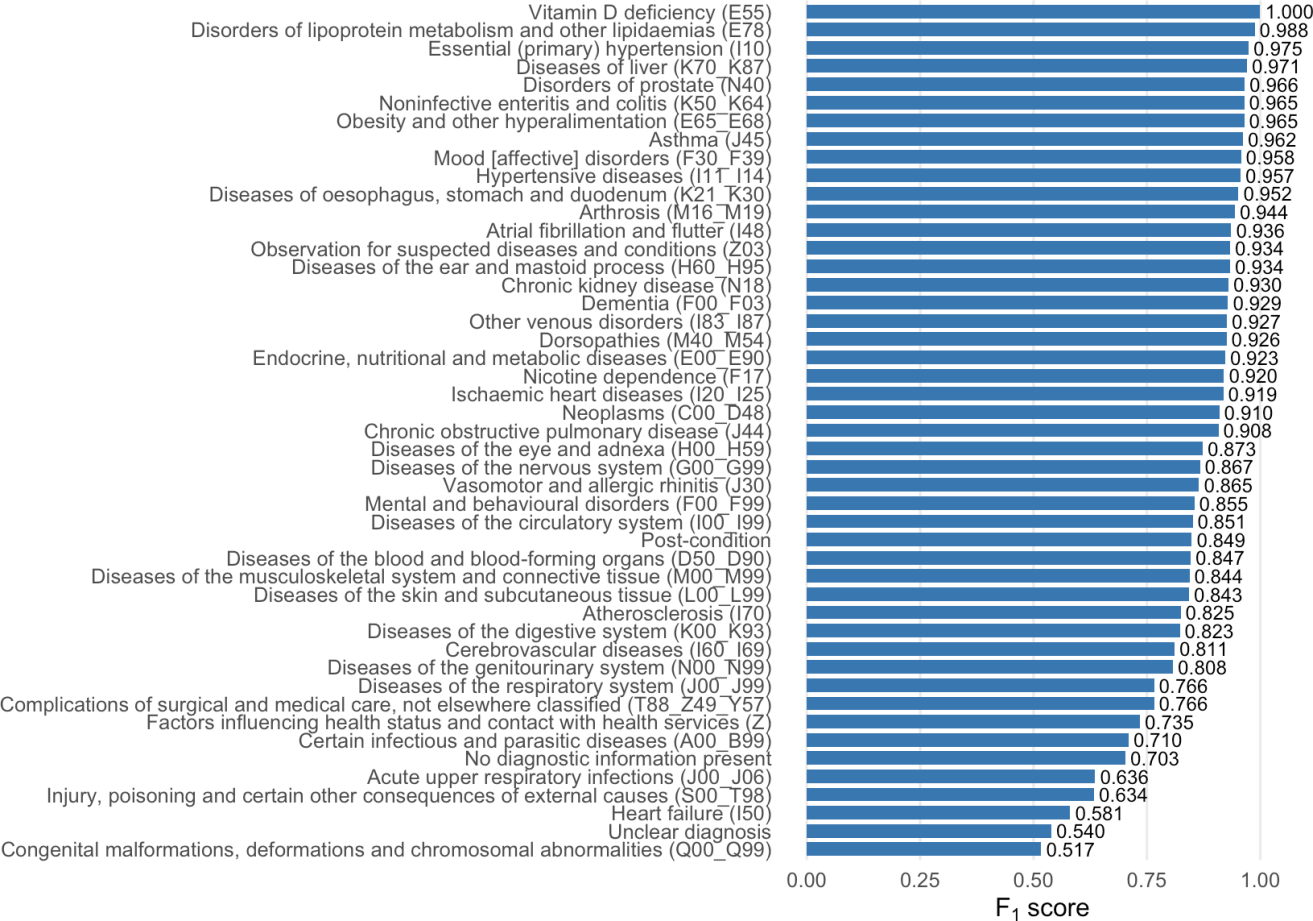
Performance of the Family Medicine Research Using Electronic Medical Records (FIRE) classifier on the posttraining test dataset. The bar chart displays the *F*_1_-scores (x-axis) for each diagnostic class (y-axis) predicted by the FIRE classifier on the 15% test posttraining dataset, unseen during training. Larger bars indicate better classification performance.

Across 47 classes, the FIRE classifier achieved strong performance on the held-out posttraining set, with *F*_1_-scores of 0.85 (micro, overall across all predictions), 0.86 (macro, mean of per-class scores treating classes equally), and 0.84 (weighted, per-class scores weighted by class frequency). The classifier achieved the highest performance for “Vitamin D deficiency” (E55) with an *F*_1_-score of 1.0, followed closely by “Disorders of lipoprotein metabolism and other lipidaemias” (E78), “Essential (primary) hypertension” (I10), and “Diseases of liver” (K70-K87), all exceeding 0.97. Strong results were also observed for a range of chronic and metabolic conditions such as obesity (E65-E68), “Asthma” (J45), and “Mood disorders” (F30-F39), with *F*_1_-scores above 0.95. In contrast, performance declined for certain classes with fewer samples or greater diagnostic overlap, such as “Unclear diagnosis” (*F*_1_-score 0.54) and “Congenital malformations, deformations and chromosomal abnormalities” (Q00-Q99; *F*_1_-score 0.52). As displayed in Figure 2, *F*_1_-scores for the posttraining test data were generally high, with variation across the different labels.

## Discussion

Extracting medical diagnoses or encounters from free-text EMRs or research data still poses critical practice challenges. By using a labeled dataset of ~53,000 records from a nationwide general practice research database, our study developed a classifier to predict appropriate codes in German free-text notes using a locally adapted diagnosis and medical encounter classification system. As such, our study offers insights into not only the challenges but also the potential rewards of creating locally adapted multiclassifier systems for medical free-text notes. Our multilabel classifier showed robust performance in classifying 47 different diagnostic and medical encounter categories during training and on unseen posttraining test data, with varying amounts of available labeled training data across categories. Our multilabel classifier was able to predict around half of the included diagnostic and medical encounter categories with high *F*_1_-scores of 0.8 or greater. These findings showcase the potential usefulness of our classifier approach for systematic use in research.

Therefore, our study demonstrates the feasibility for health care providers to develop locally adapted text classification models designed to meet their unique data requirements and integration needs within operational workflows. We encountered challenges previously discussed in the literature [11], such as predicting a large number of classes simultaneously (a large label space) and dealing with an unbalanced label distribution, in particular with the predominant class “factors influencing health status and contact with health services.” Despite preprocessing to mitigate its overrepresentation by removing free-text notes labeled with this class label, its high prevalence persisted due to co-occurrence with other classes, which we did not wish to thin out. This feature, while not ideal, reflects the real-world data scenario and underpins the relevance of our findings. Importantly, despite these real-world challenges, our classifier maintained commendable performance on both common and rare classes, as well as on new, unseen data, providing valuable insights for health care providers interested in developing custom models amid similar data challenges.

Determining the optimal sample size for training multilabel classifiers remains a topic of debate, as there is no straightforward method such as the power analysis used in hypothesis testing. Our study shows that even for low-frequency classes (ie, with fewer than 100 labeled free-text notes available) such as hypertensive diseases, model performance can still be high. For health care providers looking to develop their own classifiers, our results suggest that the required sample size for each category could be fewer than 100 text samples when training a multilabel classifier of a similar size. However, it’s important to note that the required sample size varies depending on how well the classes are defined by specific terms, such as “dementia,” versus more ambiguous terms, such as “suspected diagnosis.” Generally, our manual error investigation suggested that the quality of data labeling is of crucial importance.

There has been ongoing discussion about shifting toward prompt-based classification and moving beyond manually curated training data by leveraging industry-developed, general-purpose LLMs such as Google’s Gemini or OpenAI’s ChatGPT. However, a key drawback of currently available powerful general-purpose LLMs, such as OpenAI’s GPT-4o, is that they are proprietary, closed-source, and tied to cloud-based paid services operated by large technology companies. This makes them unsuitable for sensitive clinical data, where local processing and data privacy are essential. Nonetheless, in the future, open-source general-purpose LLMs that can be run on local infrastructure are very likely to become more capable.

Our research has several limitations that merit consideration. We used a dataset specifically designed for this use case, which is characterized by its short text notes. Documents of different types, especially those with more extensive content such as admission or discharge summaries, might require alternative methods. For example, researchers dealing with longer documents may need to use models such as Longformer [12], which are designed to process longer text data. Most transformer-based models, including German BERT, typically have a default input constraint of 128 tokens, which can be extended to a maximum of 512 tokens—any text data longer than this is omitted. In the context of the German BERT tokenizer, a token is generally equivalent to a single word. Consequently, training LLMs on longer documents using Longformer models [12] could lead to different results. In addition, the text data was manually labeled by medical school graduates, and labeling may be prone to errors. Errors in data labeling can affect model performance and are a common challenge in the data labeling process. Finally, the predictive results of the model trained in this study are specifically tailored for use at the Institute of Primary Care.

An important consideration when interpreting our results is the *ICD-10* code range Z00-Z99 “Factors influencing health status and contact with health services,” which primarily captures contextual or encounter-related information rather than explicit disease entities. In routine primary care documentation, such codes may function as a nonspecific “catch-all” category, particularly when diagnostic certainty is low. To mitigate potential bias, we excluded notes labeled exclusively with these encounter-related categories and retained them only when they co-occurred with disease-related labels, treating them as contextual modifiers rather than stand-alone outcomes. Nonetheless, its continued prevalence reflects real-world documentation practices. Notably, macro- and microaveraged *F*_1_-scores were very similar, suggesting that performance was not driven solely by the dominant encounter-related class. Accordingly, overall performance should be interpreted with an emphasis on macroaveraged metrics and alongside per-class results (Figure 2 and Multimedia Appendix 1, which contains the executed analysis and full per-class results), providing a more balanced view across both common encounter codes and less frequent disease categories. Moreover, predictions of encounter-related codes should be interpreted as reflecting health care use context rather than disease burden, and the classifier should be viewed as a screening tool rather than an autonomous diagnostic system.

Beyond these methodological considerations, an important ethical issue is the potential for residual bias amplification given the imbalanced category distribution, mainly due to the overrepresented encounter-related codes. Although we excluded free-text notes labeled exclusively with encounter-related codes, the high prevalence of this class may still influence model behavior. For this reason, transparency regarding model design, training data, and class-specific performance is essential to support responsible use and clinician adoption. The classifier presented in this study is therefore best suited for screening and structuring heterogeneous clinical free-text notes, rather than as an autonomous diagnostic system, and it should not be used in settings requiring very high precision, such as downstream epidemiological analyses. Future research should focus on improving model explainability to support clinical acceptance and to facilitate the identification of potential biases during deployment.

This study uses supervised multilabel classification, which requires a sufficient number of positive examples per label to learn stable decision boundaries and produce reliable, interpretable performance estimates. We therefore focus on more prevalent *ICD-10* classes. Diagnostic categories with very low prevalence fall into a few-shot or zero-shot regime and cannot be robustly modeled within the same supervised fine-tuning framework. Although we explored prompt-based zero-shot label assignment using an open-source German LLM, performance was inconsistent and substantially inferior to supervised classification. Accordingly, this study concentrates on diagnostic categories with adequate training data for robust supervised model development. Rare conditions remain an important but distinct methodological challenge and may be better addressed through alternative paradigms such as few-shot retrieval-based methods or entailment-based classification.

For classification, we used *bert-base-german-uncased* as a well-established and widely used baseline model to demonstrate supervised multilabel classification of German free-text notes in primary care. While alternative transformer architectures may offer performance gains, particularly through domain adaptation or different pretraining objectives, systematic comparisons were beyond the scope of this work. Relevant candidates for future evaluation include domain-adapted German biomedical models such as medBERT.de [13], GottBERT [14], and Me-LLaMA 70B [15], as well as alternative pretraining paradigms such as German ELECTRA [16], which has shown strong performance in text classification tasks. In addition, multilingual architectures (eg, the multilingual mBERT [17]) may be of interest for cross-lingual transfer scenarios. Future research would benefit from systematically assessing trade-offs between predictive performance, computational requirements, and deployment feasibility when applying these architectures to real-world clinical settings.

In summary, our study demonstrates the feasibility and benefits of training tailored multilabel text classifiers to categorize diagnostic physician notes in primary care. Combined, our work and documentation of the classifier development process lay a solid foundation for applied researchers and health care providers to leverage their own rich datasets for sophisticated statistical analyses and rigorous research. Moreover, after further validation, such multilabel classifiers could potentially also be used for generating more meaningful, precise, and actionable feedback to the participating general practitioners and thus help them improve the quality of care for their patients. We believe that our work is particularly relevant for health care organizations that accumulate vast amounts of data over time, including rich open text records that often exceed the amount of data typically collected by large research studies in a feasible time frame and budget. For example, many inpatient clinics have extensive longitudinal data, both quantitative and qualitative, including detailed patient histories across multiple admissions. However, our work also underscores the importance of high-quality data labeling and thoughtful integration of artificial intelligence into health care workflows to meaningfully enhance patient care.

## Supplementary material

10.2196/86533 Multimedia Appendix 1 Jupyter Notebook containing the Python code and complete model training output.

## Abbreviations

BERT: bidirectional encoder representations from transformers
EMR: electronic medical record
FIRE: Family Medicine Research Using Electronic Medical Records
*ICD-10*: *International Statistical Classification of Diseases, Tenth Revision*
LLM: large language model
