# Correction: Automated ICD-10–Anchored Classification of Primary Care Text Data: Development and Evaluation of a Custom Multilabel Classifier

**DOI:** 10.2196/99873

**Published:** 2026-05-29

**Authors:** Christina Haag, Thomas Grischott, Jakob M Burgstaller, Stefan Markun, Oliver Senn, Viktor von Wyl

**Affiliations:** 1 Epidemiology, Biostatistics and Prevention Institute, University of Zurich, Hirschengraben 84, Zurich, 8001, Switzerland, 41 44 63 46380; 2 Institute for Implementation Science in Health Care, University of Zurich, Zurich, Switzerland; 3 Institute of Primary Care, University Hospital Zurich, University of Zurich, Zurich, Switzerland

In “Automated ICD-10–Anchored Classification of Primary Care Text Data: Development and Evaluation of a Custom Multilabel Classifier” [1], the authors made one correction.

Authors OS and VvW are senior authors, but attribution of their joint contribution was missing in the metadata and the Acknowledgments.

An asterisk has been added to both author names, and the following footnote has been added to the metadata:

*these authors contributed equally

Furthermore, the following sentence has been added to the Acknowledgments section to clarify the senior authorship:

*OS and VvW share joint senior authorship of this work.*

The correction will appear in the online version of the paper on the JMIR Publications website, together with the publication of this correction notice. Because this was made after submission to PubMed, PubMed Central, and other full-text repositories, the corrected article has also been resubmitted to those repositories.
