# Supplementary material for: Automated ICD-10–Anchored Classification of Primary Care Text Data: Development and Evaluation of a Custom Multilabel Classifier
Source: JMIR Med Inform. 2026 Apr 6;14:e86533. doi: 10.2196/86533 (PMC13053002; doi:10.2196/86533)
Supplement: Multimedia Appendix 1 [file medinform-v14-e86533-s001.pdf]

In [3]:
```
###############################################
#                                             #
#  Building a custom multi-label classifier   #
#                                             #
###############################################

# The below code is based on this code template provided
# by Niels Rogge : https://github.com/NielsRogge/Transformers-Tutorials/blob/
#    master/BERT/Fine_tuning_BERT_(and_friends)_for_multi_label_text_classification.ipynb

# Set cache
%env TRANSFORMERS_CACHE=/home/chhaag/data/cache
%env HF_DATASETS_CACHE=/home/chhaag/data/cache

import pandas as pd
from datasets import Dataset
from transformers import AutoModelForSequenceClassification, AutoTokenizer

# Load both files into a Hugging Face object (PATH to the directory in which the files are stored)
df_train = pd.read_excel("/data/chhaag/ICD_NLP/data/icd_df_train.xlsx")
df_test  = pd.read_excel("/data/chhaag/ICD_NLP/data/icd_df_test_data_for_training.xlsx")


# Converts pandas DataFrames into Hugging Face Dataset objects
train = Dataset.from_pandas(df_train)
test = Dataset.from_pandas(df_test)

labels = [label for label in train.features.keys() if label not in ['text']]
id2label = {idx:label for idx, label in enumerate(labels)}
label2id = {label:idx for idx, label in enumerate(labels)}
labels

print(len(labels))
print(labels)
```

```
env: TRANSFORMERS_CACHE=/home/chhaag/data/cache
env: HF_DATASETS_CACHE=/home/chhaag/data/cache
47
['A00_B99', 'C00_D48', 'D50_D90', 'E00_E90', 'E55', 'E65_E68', 'E78', 'F00_F99', 'F00_F03', 'F17', 'F30_F39', 'G00_G
99', 'H00_H59', 'H60_H95', 'I00_I99', 'I10', 'I11_I14', 'I20_I25', 'I48', 'I50', 'I60_I69', 'I70', 'I83_I87', 'J00_J
99', 'J00_J06', 'J30', 'J44', 'J45', 'K00_K93', 'K21_K30', 'K50_K64', 'K70_K87', 'L00_L99', 'M00_M99', 'M16_M19', 'M
40_M54', 'N00_N99', 'N18', 'N40', 'Q00_Q99', 'S00_T98', 'ZR', 'T88_7_X49_Y57', 'verdacht', 'unklar', 'keine', 'Stn']
```

In [4]:

```
### Preprocessing

from transformers import AutoTokenizer
import numpy as np

# Initializing the Tokenizer
tokenizer = AutoTokenizer.from_pretrained("/data/chhaag/ICD_NLP/GermanBERTuncased")


# Tokenizing the dataset and processing labels for multi-label classification

def preprocess_data(examples):
  # take a batch of texts
  text = examples["text"]
  # encode them
  encoding = tokenizer(text, padding="max_length", truncation=True, max_length=512)
  # add labels
  labels_batch = {k: examples[k] for k in examples.keys() if k in labels}
  # create numpy array of shape (batch_size, num_labels)
  labels_matrix = np.zeros((len(text), len(labels)))
  # fill numpy array
  for idx, label in enumerate(labels):
    labels_matrix[:, idx] = labels_batch[label]

  encoding["labels"] = labels_matrix.tolist()

  return encoding
```

In [5]:

```
# Apply pre-process function to training and validation data
enc_train = train.map(preprocess_data, batched=True, remove_columns=train.column_names)
enc_test  = test.map(preprocess_data, batched=True, remove_columns=test.column_names)
```

```
# Set format to PyTorch tensors
enc_train.set_format("torch")
enc_test.set_format("torch")
```

```
Map: 100%|██████████| 27108/27108 [00:06<00:00, 4295.36 examples/s]
Map: 100%|██████████| 5810/5810 [00:01<00:00, 4442.30 examples/s]
```

In [6]:

```
# Define model

from transformers import AutoModelForSequenceClassification

model = AutoModelForSequenceClassification.from_pretrained(
        "/data/chhaag/ICD_NLP/GermanBERTuncased",
        problem_type="multi_label_classification",  # ensures appropriate loss function is applied (BCEWithLogitsLo
                                                     # https://pytorch.org/docs/stable/generated/torch.nn.BCEWithLog
        num_labels=len(labels),                      # size output layer
        id2label=id2label,
        label2id=label2id)
```

```
Some weights of BertForSequenceClassification were not initialized from the model checkpoint at /data/chhaag/ICD_NL
P/GermanBERTuncased and are newly initialized: ['classifier.bias', 'classifier.weight']
You should probably TRAIN this model on a down-stream task to be able to use it for predictions and inference.
```

In [7]:

```
# Training the model

batch_size = 8
metric_name = "f1"

from transformers import TrainingArguments, Trainer

args = TrainingArguments(
    output_dir="/data/chhaag/ICD_NLP/output",
    evaluation_strategy = "epoch",
    save_strategy = "epoch",
    learning_rate=2e-5,
    per_device_train_batch_size=batch_size,
    per_device_eval_batch_size=batch_size,
    num_train_epochs=10,
    weight_decay=0.01,
    load_best_model_at_end=True,
    metric_for_best_model=metric_name,
```

```
    push_to_hub=False
)
```

```
/data/chhaag/conda/envs/myenv3/lib/python3.11/site-packages/transformers/training_args.py:1594: FutureWarning: `evaluation_strategy` is deprecated and will be removed in version 4.46 of 🤗 Transformers. Use `eval_strategy` instead
  warnings.warn(
```

In [8]:

```python
from sklearn.metrics import f1_score, roc_auc_score, accuracy_score
from sklearn.metrics import classification_report
from transformers import EvalPrediction
import torch


# source: https://jesusleal.io/2021/04/21/Longformer-multilabel-classification/
def multi_label_metrics(predictions, labels, threshold=0.5):
    # first, apply sigmoid on predictions which are of shape (batch_size, num_labels)
    sigmoid = torch.nn.Sigmoid()
    probs = sigmoid(torch.Tensor(predictions))
    # next, use threshold to turn them into integer predictions
    y_pred = np.zeros(probs.shape)
    y_pred[np.where(probs >= threshold)] = 1
    # finally, compute metrics
    y_true = labels
    f1_micro_average = f1_score(y_true=y_true, y_pred=y_pred, average='micro')
    roc_auc = roc_auc_score(y_true, y_pred, average = 'micro')
    accuracy = accuracy_score(y_true, y_pred)
    # return as dictionary
    metrics = {'f1': f1_micro_average,
               'roc_auc': roc_auc,
               'accuracy': accuracy}
    print(classification_report(y_true, y_pred))

    return metrics


def compute_metrics(p: EvalPrediction):
    preds = p.predictions[0] if isinstance(p.predictions,
            tuple) else p.predictions
    result = multi_label_metrics(
        predictions=preds,
```

```
        labels=p.label_ids)
    return result
```

In [9]:
```
# CHECK: Verifying a batch + a forward pass

# Batch
print(enc_train[0]['labels'].type())
print(enc_train['input_ids'][0])

# Forward pass
outputs = model(input_ids=enc_train['input_ids'][0].unsqueeze(0),
                labels=enc_train[0]['labels'].unsqueeze(0))
outputs
```

```
We strongly recommend passing in an `attention_mask` since your input_ids may be padded. See https://huggingface.co/
docs/transformers/troubleshooting#incorrect-output-when-padding-tokens-arent-masked.
```

```
torch.FloatTensor
tensor([ 102, 6460, 3153,  103,    0,    0,    0,    0,    0,    0,    0,    0,
           0,    0,    0,    0,    0,    0,    0,    0,    0,    0,    0,    0,
           0,    0,    0,    0,    0,    0,    0,    0,    0,    0,    0,    0,
           0,    0,    0,    0,    0,    0,    0,    0,    0,    0,    0,    0,
           0,    0,    0,    0,    0,    0,    0,    0,    0,    0,    0,    0,
           0,    0,    0,    0,    0,    0,    0,    0,    0,    0,    0,    0,
           0,    0,    0,    0,    0,    0,    0,    0,    0,    0,    0,    0,
           0,    0,    0,    0,    0,    0,    0,    0,    0,    0,    0,    0,
           0,    0,    0,    0,    0,    0,    0,    0,    0,    0,    0,    0,
           0,    0,    0,    0,    0,    0,    0,    0,    0,    0,    0,    0,
           0,    0,    0,    0,    0,    0,    0,    0,    0,    0,    0,    0,
           0,    0,    0,    0,    0,    0,    0,    0,    0,    0,    0,    0,
           0,    0,    0,    0,    0,    0,    0,    0,    0,    0,    0,    0,
           0,    0,    0,    0,    0,    0,    0,    0,    0,    0,    0,    0,
           0,    0,    0,    0,    0,    0,    0,    0,    0,    0,    0,    0,
           0,    0,    0,    0,    0,    0,    0,    0,    0,    0,    0,    0,
           0,    0,    0,    0,    0,    0,    0,    0,    0,    0,    0,    0,
           0,    0,    0,    0,    0,    0,    0,    0,    0,    0,    0,    0,
           0,    0,    0,    0,    0,    0,    0,    0,    0,    0,    0,    0,
           0,    0,    0,    0,    0,    0,    0,    0,    0,    0,    0,    0,
           0,    0,    0,    0,    0,    0,    0,    0,    0,    0,    0,    0,
           0,    0,    0,    0,    0,    0,    0,    0,    0,    0,    0,    0,
           0,    0,    0,    0,    0,    0,    0,    0,    0,    0,    0,    0,
           0,    0,    0,    0,    0,    0,    0,    0,    0,    0,    0,    0,
           0,    0,    0,    0,    0,    0,    0,    0,    0,    0,    0,    0,
           0,    0,    0,    0,    0,    0,    0,    0,    0,    0,    0,    0,
           0,    0,    0,    0,    0,    0,    0,    0,    0,    0,    0,    0,
           0,    0,    0,    0,    0,    0,    0,    0,    0,    0,    0,    0,
           0,    0,    0,    0,    0,    0,    0,    0,    0,    0,    0,    0,
           0,    0,    0,    0,    0,    0,    0,    0,    0,    0,    0,    0,
           0,    0,    0,    0,    0,    0,    0,    0,    0,    0,    0,    0,
           0,    0,    0,    0,    0,    0,    0,    0,    0,    0,    0,    0,
           0,    0,    0,    0,    0,    0,    0,    0,    0,    0,    0,    0,
           0,    0,    0,    0,    0,    0,    0,    0,    0,    0,    0,    0,
           0,    0,    0,    0,    0,    0,    0,    0,    0,    0,    0,    0,
           0,    0,    0,    0,    0,    0,    0,    0,    0,    0,    0,    0,
           0,    0,    0,    0,    0,    0,    0,    0,    0,    0,    0,    0,
           0,    0,    0,    0,    0,    0,    0,    0,    0,    0,    0,    0,
           0,    0,    0,    0,    0,    0,    0,    0,    0,    0,    0,    0,
           0,    0,    0,    0,    0,    0,    0,    0,    0,    0,    0,    0,
```

```
        0,    0,    0,    0,    0,    0,    0,    0,    0,    0,    0,    0,
        0,    0,    0,    0,    0,    0,    0,    0,    0,    0,    0,    0,
        0,    0,    0,    0,    0,    0,    0,    0])
```

Out[9]:

```
SequenceClassifierOutput(loss=tensor(0.7553, grad_fn=<BinaryCrossEntropyWithLogitsBackward0>), logits=tensor([[-0.
0465,  0.2431, -0.5856, -0.4254, -0.2144, -0.2093, -0.6289,  0.2723,
          0.2885, -0.1950,  0.0957,  0.1468, -0.1019, -0.0124,  0.0042,  0.1828,
         -0.0952,  0.8050,  0.3709,  0.8313,  0.3440, -0.4556,  0.1552,  0.1128,
          0.5118, -0.2391,  0.2627,  0.6727,  0.0542,  0.4196,  0.1928, -0.1138,
         -0.9235,  0.1885,  0.0617,  0.4720,  0.4042, -0.1311, -0.4190,  0.5422,
          0.0473,  0.4294,  0.4669,  0.4717, -0.0728, -0.2898,  0.5199]],
       grad_fn=<AddmmBackward0>), hidden_states=None, attentions=None)
```

In [10]:

```
# Start model training

trainer = Trainer(
    model,
    args,
    train_dataset=enc_train,
    eval_dataset=enc_test,
    tokenizer=tokenizer,
    compute_metrics=compute_metrics,
)
```

```
/sctmp/chhaag/ipykernel_3910311/742856707.py:3: FutureWarning: `tokenizer` is deprecated and will be removed in vers
ion 5.0.0 for `Trainer.__init__`. Use `processing_class` instead.
  trainer = Trainer(
```

In [11]:

```
# Run model training

trainer.train()
```

[33890/33890 1:38:35, Epoch 10/10]

Epoch	Training Loss	Validation Loss	F1	Roc Auc	Accuracy
1	0.053900	0.044299	0.696026	0.784483	0.530637
2	0.032100	0.030971	0.819722	0.893295	0.697590
3	0.023200	0.026886	0.831745	0.894073	0.715835
4	0.017300	0.026272	0.841352	0.909021	0.731153
5	0.013000	0.026036	0.844821	0.911662	0.739587
6	0.009800	0.027034	0.847011	0.916262	0.741136
7	0.007700	0.028422	0.842663	0.905666	0.737694
8	0.006000	0.028887	0.848408	0.915840	0.743029
9	0.004500	0.029154	0.848130	0.918497	0.739931
10	0.003800	0.029342	0.848844	0.914787	0.743201

	precision	recall	f1-score	support
0	0.00	0.00	0.00	92
1	0.89	0.76	0.82	284
2	1.00	0.19	0.32	74
3	0.92	0.88	0.90	319
4	1.00	0.66	0.79	32
5	1.00	0.85	0.92	85
6	0.97	0.99	0.98	173
7	0.95	0.54	0.69	148
8	0.00	0.00	0.00	14
9	0.86	0.44	0.58	57
10	0.97	0.86	0.91	84
11	0.90	0.63	0.74	234
12	0.96	0.20	0.33	110
13	0.98	0.60	0.74	84
14	0.85	0.66	0.74	265
15	0.95	0.98	0.96	258
16	1.00	0.09	0.16	46
17	0.88	0.42	0.57	90
18	1.00	0.81	0.90	48
19	0.00	0.00	0.00	17
20	0.00	0.00	0.00	37
21	0.00	0.00	0.00	32
22	0.98	0.68	0.80	80
23	0.00	0.00	0.00	74
24	0.00	0.00	0.00	25
25	1.00	0.03	0.05	37
26	0.00	0.00	0.00	25
27	0.93	0.77	0.84	65
28	0.98	0.31	0.47	184
29	0.90	0.84	0.87	123
30	0.98	0.88	0.92	153
31	0.00	0.00	0.00	53
32	0.94	0.41	0.57	140
33	0.89	0.73	0.80	432
34	0.92	0.87	0.89	117
35	0.89	0.92	0.91	318
36	0.91	0.13	0.22	157
37	1.00	0.63	0.77	43
38	1.00	0.64	0.78	42

```
          39       0.00      0.00      0.00        37
          40       0.00      0.00      0.00        59
          41       0.78      0.46      0.58      1234
          42       1.00      0.03      0.05        71
          43       0.94      0.87      0.91       208
          44       0.00      0.00      0.00       231
          45       0.91      0.37      0.52       187
          46       0.83      0.71      0.77       548

   micro avg       0.89      0.57      0.70      7226
   macro avg       0.70      0.44      0.51      7226
weighted avg       0.81      0.57      0.64      7226
 samples avg       0.64      0.57      0.59      7226
```

```
/data/chhaag/conda/envs/myenv3/lib/python3.11/site-packages/sklearn/metrics/_classification.py:1565: UndefinedMetric
Warning: Precision is ill-defined and being set to 0.0 in labels with no predicted samples. Use `zero_division` para
meter to control this behavior.
  _warn_prf(average, modifier, f"{metric.capitalize()} is", len(result))
/data/chhaag/conda/envs/myenv3/lib/python3.11/site-packages/sklearn/metrics/_classification.py:1565: UndefinedMetric
Warning: Precision is ill-defined and being set to 0.0 in samples with no predicted labels. Use `zero_division` para
meter to control this behavior.
  _warn_prf(average, modifier, f"{metric.capitalize()} is", len(result))
/data/chhaag/conda/envs/myenv3/lib/python3.11/site-packages/sklearn/metrics/_classification.py:1565: UndefinedMetric
Warning: Recall is ill-defined and being set to 0.0 in samples with no true labels. Use `zero_division` parameter to
control this behavior.
  _warn_prf(average, modifier, f"{metric.capitalize()} is", len(result))
/data/chhaag/conda/envs/myenv3/lib/python3.11/site-packages/sklearn/metrics/_classification.py:1565: UndefinedMetric
Warning: F-score is ill-defined and being set to 0.0 in samples with no true nor predicted labels. Use `zero_divisio
n` parameter to control this behavior.
  _warn_prf(average, modifier, f"{metric.capitalize()} is", len(result))
```

```
              precision    recall  f1-score   support

           0       0.74      0.32      0.44        92
           1       0.83      0.87      0.85       284
           2       0.96      0.73      0.83        74
           3       0.94      0.87      0.90       319
           4       0.97      1.00      0.98        32
           5       1.00      0.91      0.95        85
           6       0.98      0.99      0.98       173
           7       0.79      0.86      0.83       148
           8       1.00      0.71      0.83        14
           9       0.93      0.75      0.83        57
          10       0.99      0.90      0.94        84
          11       0.80      0.80      0.80       234
          12       0.87      0.79      0.83       110
          13       0.88      0.81      0.84        84
          14       0.84      0.79      0.82       265
          15       0.98      0.97      0.98       258
          16       0.91      0.93      0.92        46
          17       0.80      0.93      0.86        90
          18       1.00      0.98      0.99        48
          19       0.00      0.00      0.00        17
          20       0.73      0.22      0.33        37
          21       0.95      0.56      0.71        32
          22       0.92      0.88      0.90        80
          23       0.75      0.64      0.69        74
          24       1.00      0.20      0.33        25
          25       0.88      0.97      0.92        37
          26       0.90      0.76      0.83        25
          27       0.94      1.00      0.97        65
          28       0.86      0.70      0.77       184
          29       0.90      0.91      0.90       123
          30       0.97      0.93      0.95       153
          31       0.92      0.68      0.78        53
          32       0.88      0.65      0.75       140
          33       0.89      0.84      0.86       432
          34       0.92      0.92      0.92       117
          35       0.90      0.96      0.93       318
          36       0.80      0.69      0.74       157
          37       0.87      0.93      0.90        43
          38       0.93      0.98      0.95        42
```

```
          39       0.00      0.00      0.00        37
          40       0.92      0.61      0.73        59
          41       0.72      0.79      0.75      1234
          42       0.90      0.51      0.65        71
          43       0.95      0.93      0.94       208
          44       0.66      0.26      0.37       231
          45       0.80      0.66      0.72       187
          46       0.86      0.78      0.82       548

   micro avg       0.85      0.79      0.82      7226
   macro avg       0.85      0.74      0.78      7226
weighted avg       0.84      0.79      0.81      7226
 samples avg       0.78      0.76      0.76      7226
```

```
/data/chhaag/conda/envs/myenv3/lib/python3.11/site-packages/sklearn/metrics/_classification.py:1565: UndefinedMetric
Warning: Precision is ill-defined and being set to 0.0 in labels with no predicted samples. Use `zero_division` para
meter to control this behavior.
  _warn_prf(average, modifier, f"{metric.capitalize()} is", len(result))
/data/chhaag/conda/envs/myenv3/lib/python3.11/site-packages/sklearn/metrics/_classification.py:1565: UndefinedMetric
Warning: Precision is ill-defined and being set to 0.0 in samples with no predicted labels. Use `zero_division` para
meter to control this behavior.
  _warn_prf(average, modifier, f"{metric.capitalize()} is", len(result))
/data/chhaag/conda/envs/myenv3/lib/python3.11/site-packages/sklearn/metrics/_classification.py:1565: UndefinedMetric
Warning: Recall is ill-defined and being set to 0.0 in samples with no true labels. Use `zero_division` parameter to
control this behavior.
  _warn_prf(average, modifier, f"{metric.capitalize()} is", len(result))
/data/chhaag/conda/envs/myenv3/lib/python3.11/site-packages/sklearn/metrics/_classification.py:1565: UndefinedMetric
Warning: F-score is ill-defined and being set to 0.0 in samples with no true nor predicted labels. Use `zero_divisio
n` parameter to control this behavior.
  _warn_prf(average, modifier, f"{metric.capitalize()} is", len(result))
```

	precision	recall	f1-score	support
0	0.71	0.54	0.62	92
1	0.89	0.87	0.88	284
2	0.87	0.84	0.86	74
3	0.94	0.89	0.92	319
4	0.97	0.88	0.92	32
5	0.99	0.93	0.96	85
6	0.99	0.97	0.98	173
7	0.93	0.77	0.84	148
8	1.00	0.71	0.83	14
9	0.96	0.81	0.88	57
10	0.99	0.88	0.93	84
11	0.89	0.76	0.82	234
12	0.88	0.79	0.83	110
13	0.92	0.80	0.85	84
14	0.90	0.76	0.82	265
15	0.98	0.96	0.97	258
16	0.93	0.93	0.93	46
17	0.89	0.86	0.87	90
18	1.00	0.96	0.98	48
19	0.00	0.00	0.00	17
20	0.74	0.38	0.50	37
21	0.95	0.59	0.73	32
22	0.90	0.90	0.90	80
23	0.83	0.59	0.69	74
24	0.89	0.32	0.47	25
25	0.94	0.92	0.93	37
26	0.95	0.84	0.89	25
27	0.96	1.00	0.98	65
28	0.86	0.71	0.78	184
29	0.91	0.93	0.92	123
30	0.98	0.93	0.96	153
31	0.91	0.81	0.86	53
32	0.85	0.71	0.78	140
33	0.91	0.82	0.86	432
34	0.93	0.95	0.94	117
35	0.93	0.93	0.93	318
36	0.82	0.68	0.75	157
37	1.00	0.93	0.96	43
38	0.87	0.98	0.92	42

```
          39       0.00      0.00      0.00        37
          40       0.91      0.68      0.78        59
          41       0.77      0.73      0.75      1234
          42       0.82      0.59      0.69        71
          43       0.96      0.94      0.95       208
          44       0.62      0.39      0.48       231
          45       0.82      0.68      0.74       187
          46       0.86      0.82      0.84       548

   micro avg       0.88      0.79      0.83      7226
   macro avg       0.86      0.76      0.80      7226
weighted avg       0.87      0.79      0.82      7226
 samples avg       0.79      0.77      0.77      7226
```

```
/data/chhaag/conda/envs/myenv3/lib/python3.11/site-packages/sklearn/metrics/_classification.py:1565: UndefinedMetric
Warning: Precision is ill-defined and being set to 0.0 in labels with no predicted samples. Use `zero_division` para
meter to control this behavior.
  _warn_prf(average, modifier, f"{metric.capitalize()} is", len(result))
/data/chhaag/conda/envs/myenv3/lib/python3.11/site-packages/sklearn/metrics/_classification.py:1565: UndefinedMetric
Warning: Precision is ill-defined and being set to 0.0 in samples with no predicted labels. Use `zero_division` para
meter to control this behavior.
  _warn_prf(average, modifier, f"{metric.capitalize()} is", len(result))
/data/chhaag/conda/envs/myenv3/lib/python3.11/site-packages/sklearn/metrics/_classification.py:1565: UndefinedMetric
Warning: Recall is ill-defined and being set to 0.0 in samples with no true labels. Use `zero_division` parameter to
control this behavior.
  _warn_prf(average, modifier, f"{metric.capitalize()} is", len(result))
/data/chhaag/conda/envs/myenv3/lib/python3.11/site-packages/sklearn/metrics/_classification.py:1565: UndefinedMetric
Warning: F-score is ill-defined and being set to 0.0 in samples with no true nor predicted labels. Use `zero_divisio
n` parameter to control this behavior.
  _warn_prf(average, modifier, f"{metric.capitalize()} is", len(result))
```

	precision	recall	f1-score	support
0	0.77	0.52	0.62	92
1	0.89	0.87	0.88	284
2	0.89	0.84	0.86	74
3	0.94	0.92	0.93	319
4	0.97	0.88	0.92	32
5	1.00	0.94	0.97	85
6	0.99	1.00	1.00	173
7	0.93	0.82	0.87	148
8	1.00	0.79	0.88	14
9	0.83	0.88	0.85	57
10	0.96	0.94	0.95	84
11	0.86	0.81	0.83	234
12	0.90	0.82	0.86	110
13	0.91	0.85	0.88	84
14	0.91	0.81	0.86	265
15	0.98	0.97	0.97	258
16	0.96	0.93	0.95	46
17	0.94	0.83	0.88	90
18	1.00	0.96	0.98	48
19	1.00	0.18	0.30	17
20	0.76	0.70	0.73	37
21	1.00	0.59	0.75	32
22	0.89	0.89	0.89	80
23	0.80	0.70	0.75	74
24	1.00	0.24	0.39	25
25	0.95	0.97	0.96	37
26	0.88	0.92	0.90	25
27	0.94	1.00	0.97	65
28	0.80	0.76	0.78	184
29	0.94	0.94	0.94	123
30	0.97	0.95	0.96	153
31	0.90	0.87	0.88	53
32	0.85	0.71	0.77	140
33	0.84	0.88	0.86	432
34	0.91	0.96	0.93	117
35	0.92	0.95	0.94	318
36	0.78	0.75	0.77	157
37	1.00	0.95	0.98	43
38	0.91	0.95	0.93	42

```
          39       1.00      0.05      0.10        37
          40       0.89      0.69      0.78        59
          41       0.74      0.79      0.76      1234
          42       0.71      0.68      0.69        71
          43       0.95      0.96      0.95       208
          44       0.62      0.37      0.47       231
          45       0.80      0.67      0.73       187
          46       0.88      0.82      0.85       548

   micro avg       0.86      0.82      0.84      7226
   macro avg       0.90      0.79      0.82      7226
weighted avg       0.86      0.82      0.84      7226
 samples avg       0.80      0.79      0.79      7226
```

```
/data/chhaag/conda/envs/myenv3/lib/python3.11/site-packages/sklearn/metrics/_classification.py:1565: UndefinedMetric
Warning: Precision is ill-defined and being set to 0.0 in samples with no predicted labels. Use `zero_division` para
meter to control this behavior.
  _warn_prf(average, modifier, f"{metric.capitalize()} is", len(result))
/data/chhaag/conda/envs/myenv3/lib/python3.11/site-packages/sklearn/metrics/_classification.py:1565: UndefinedMetric
Warning: Recall is ill-defined and being set to 0.0 in samples with no true labels. Use `zero_division` parameter to
control this behavior.
  _warn_prf(average, modifier, f"{metric.capitalize()} is", len(result))
/data/chhaag/conda/envs/myenv3/lib/python3.11/site-packages/sklearn/metrics/_classification.py:1565: UndefinedMetric
Warning: F-score is ill-defined and being set to 0.0 in samples with no true nor predicted labels. Use `zero_divisio
n` parameter to control this behavior.
  _warn_prf(average, modifier, f"{metric.capitalize()} is", len(result))
```

	precision	recall	f1-score	support
0	0.79	0.58	0.67	92
1	0.87	0.89	0.88	284
2	0.88	0.86	0.87	74
3	0.91	0.94	0.92	319
4	0.97	1.00	0.98	32
5	1.00	0.94	0.97	85
6	0.99	1.00	1.00	173
7	0.87	0.84	0.86	148
8	1.00	0.79	0.88	14
9	0.96	0.79	0.87	57
10	0.99	0.93	0.96	84
11	0.83	0.83	0.83	234
12	0.88	0.84	0.86	110
13	0.90	0.86	0.88	84
14	0.92	0.77	0.84	265
15	0.98	0.98	0.98	258
16	0.95	0.91	0.93	46
17	0.94	0.81	0.87	90
18	1.00	0.94	0.97	48
19	1.00	0.29	0.45	17
20	0.78	0.68	0.72	37
21	0.87	0.62	0.73	32
22	0.90	0.91	0.91	80
23	0.77	0.74	0.76	74
24	0.77	0.40	0.53	25
25	0.95	0.95	0.95	37
26	0.89	0.96	0.92	25
27	0.94	1.00	0.97	65
28	0.87	0.76	0.81	184
29	0.92	0.96	0.94	123
30	0.99	0.92	0.95	153
31	0.92	0.83	0.87	53
32	0.80	0.76	0.78	140
33	0.87	0.87	0.87	432
34	0.92	0.92	0.92	117
35	0.94	0.96	0.95	318
36	0.79	0.79	0.79	157
37	1.00	0.95	0.98	43
38	0.93	0.98	0.95	42

```
          39       0.75      0.32      0.45        37
          40       0.86      0.73      0.79        59
          41       0.77      0.74      0.76      1234
          42       0.82      0.66      0.73        71
          43       0.94      0.95      0.94       208
          44       0.57      0.53      0.55       231
          45       0.82      0.66      0.73       187
          46       0.84      0.86      0.85       548

   micro avg       0.86      0.83      0.84      7226
   macro avg       0.89      0.81      0.84      7226
weighted avg       0.86      0.83      0.84      7226
 samples avg       0.81      0.80      0.79      7226
```

```
/data/chhaag/conda/envs/myenv3/lib/python3.11/site-packages/sklearn/metrics/_classification.py:1565: UndefinedMetric
Warning: Precision is ill-defined and being set to 0.0 in samples with no predicted labels. Use `zero_division` para
meter to control this behavior.
  _warn_prf(average, modifier, f"{metric.capitalize()} is", len(result))
/data/chhaag/conda/envs/myenv3/lib/python3.11/site-packages/sklearn/metrics/_classification.py:1565: UndefinedMetric
Warning: Recall is ill-defined and being set to 0.0 in samples with no true labels. Use `zero_division` parameter to
control this behavior.
  _warn_prf(average, modifier, f"{metric.capitalize()} is", len(result))
/data/chhaag/conda/envs/myenv3/lib/python3.11/site-packages/sklearn/metrics/_classification.py:1565: UndefinedMetric
Warning: F-score is ill-defined and being set to 0.0 in samples with no true nor predicted labels. Use `zero_divisio
n` parameter to control this behavior.
  _warn_prf(average, modifier, f"{metric.capitalize()} is", len(result))
```

	precision	recall	f1-score	support
0	0.79	0.62	0.70	92
1	0.88	0.87	0.87	284
2	0.88	0.88	0.88	74
3	0.94	0.94	0.94	319
4	0.97	1.00	0.98	32
5	1.00	0.95	0.98	85
6	0.99	1.00	0.99	173
7	0.91	0.84	0.87	148
8	1.00	0.79	0.88	14
9	0.92	0.86	0.89	57
10	0.99	0.92	0.95	84
11	0.84	0.82	0.83	234
12	0.91	0.80	0.85	110
13	0.91	0.83	0.87	84
14	0.89	0.83	0.86	265
15	0.98	0.98	0.98	258
16	0.96	0.93	0.95	46
17	0.92	0.88	0.90	90
18	1.00	0.96	0.98	48
19	1.00	0.59	0.74	17
20	0.81	0.70	0.75	37
21	0.83	0.59	0.69	32
22	0.88	0.91	0.90	80
23	0.79	0.73	0.76	74
24	1.00	0.52	0.68	25
25	0.88	0.95	0.91	37
26	0.89	0.96	0.92	25
27	0.94	1.00	0.97	65
28	0.80	0.80	0.80	184
29	0.94	0.97	0.95	123
30	0.97	0.97	0.97	153
31	0.87	0.87	0.87	53
32	0.86	0.69	0.77	140
33	0.90	0.84	0.87	432
34	0.92	0.96	0.94	117
35	0.92	0.96	0.94	318
36	0.77	0.72	0.75	157
37	1.00	0.93	0.96	43
38	0.91	0.98	0.94	42

```
          39       0.73      0.30      0.42        37
          40       0.81      0.75      0.78        59
          41       0.74      0.80      0.77      1234
          42       0.84      0.69      0.76        71
          43       0.96      0.94      0.95       208
          44       0.59      0.52      0.55       231
          45       0.74      0.73      0.73       187
          46       0.87      0.81      0.84       548

   micro avg       0.86      0.84      0.85      7226
   macro avg       0.89      0.83      0.85      7226
weighted avg       0.86      0.84      0.85      7226
 samples avg       0.81      0.80      0.79      7226
```

```
/data/chhaag/conda/envs/myenv3/lib/python3.11/site-packages/sklearn/metrics/_classification.py:1565: UndefinedMetric
Warning: Precision is ill-defined and being set to 0.0 in samples with no predicted labels. Use `zero_division` para
meter to control this behavior.
  _warn_prf(average, modifier, f"{metric.capitalize()} is", len(result))
/data/chhaag/conda/envs/myenv3/lib/python3.11/site-packages/sklearn/metrics/_classification.py:1565: UndefinedMetric
Warning: Recall is ill-defined and being set to 0.0 in samples with no true labels. Use `zero_division` parameter to
control this behavior.
  _warn_prf(average, modifier, f"{metric.capitalize()} is", len(result))
/data/chhaag/conda/envs/myenv3/lib/python3.11/site-packages/sklearn/metrics/_classification.py:1565: UndefinedMetric
Warning: F-score is ill-defined and being set to 0.0 in samples with no true nor predicted labels. Use `zero_divisio
n` parameter to control this behavior.
  _warn_prf(average, modifier, f"{metric.capitalize()} is", len(result))
```

```
              precision    recall  f1-score   support

           0       0.73      0.64      0.68        92
           1       0.91      0.84      0.87       284
           2       0.88      0.85      0.86        74
           3       0.93      0.93      0.93       319
           4       0.97      0.97      0.97        32
           5       0.99      0.95      0.97        85
           6       0.99      1.00      1.00       173
           7       0.90      0.80      0.85       148
           8       1.00      0.86      0.92        14
           9       0.91      0.84      0.87        57
          10       0.99      0.93      0.96        84
          11       0.86      0.84      0.85       234
          12       0.88      0.84      0.86       110
          13       0.90      0.88      0.89        84
          14       0.90      0.84      0.87       265
          15       0.98      0.97      0.98       258
          16       0.94      0.96      0.95        46
          17       0.92      0.86      0.89        90
          18       1.00      0.96      0.98        48
          19       1.00      0.65      0.79        17
          20       0.75      0.73      0.74        37
          21       0.83      0.62      0.71        32
          22       0.91      0.90      0.91        80
          23       0.88      0.68      0.76        74
          24       0.78      0.84      0.81        25
          25       0.90      0.95      0.92        37
          26       0.89      1.00      0.94        25
          27       0.94      1.00      0.97        65
          28       0.83      0.78      0.81       184
          29       0.92      0.97      0.94       123
          30       0.98      0.94      0.96       153
          31       0.89      0.89      0.89        53
          32       0.80      0.76      0.78       140
          33       0.85      0.86      0.86       432
          34       0.92      0.91      0.91       117
          35       0.93      0.96      0.94       318
          36       0.76      0.77      0.77       157
          37       1.00      0.95      0.98        43
          38       0.93      0.98      0.95        42
```

```
          39       0.65      0.41      0.50        37
          40       0.82      0.76      0.79        59
          41       0.81      0.66      0.72      1234
          42       0.78      0.70      0.74        71
          43       0.95      0.96      0.96       208
          44       0.62      0.44      0.51       231
          45       0.82      0.72      0.76       187
          46       0.85      0.83      0.84       548

   micro avg       0.87      0.81      0.84      7226
   macro avg       0.88      0.84      0.86      7226
weighted avg       0.87      0.81      0.84      7226
 samples avg       0.81      0.79      0.79      7226
```

```
/data/chhaag/conda/envs/myenv3/lib/python3.11/site-packages/sklearn/metrics/_classification.py:1565: UndefinedMetric
Warning: Precision is ill-defined and being set to 0.0 in samples with no predicted labels. Use `zero_division` para
meter to control this behavior.
  _warn_prf(average, modifier, f"{metric.capitalize()} is", len(result))
/data/chhaag/conda/envs/myenv3/lib/python3.11/site-packages/sklearn/metrics/_classification.py:1565: UndefinedMetric
Warning: Recall is ill-defined and being set to 0.0 in samples with no true labels. Use `zero_division` parameter to
control this behavior.
  _warn_prf(average, modifier, f"{metric.capitalize()} is", len(result))
/data/chhaag/conda/envs/myenv3/lib/python3.11/site-packages/sklearn/metrics/_classification.py:1565: UndefinedMetric
Warning: F-score is ill-defined and being set to 0.0 in samples with no true nor predicted labels. Use `zero_divisio
n` parameter to control this behavior.
  _warn_prf(average, modifier, f"{metric.capitalize()} is", len(result))
```

	precision	recall	f1-score	support
0	0.72	0.67	0.70	92
1	0.89	0.86	0.87	284
2	0.84	0.89	0.86	74
3	0.93	0.93	0.93	319
4	0.97	0.97	0.97	32
5	1.00	0.94	0.97	85
6	0.99	1.00	1.00	173
7	0.87	0.83	0.85	148
8	1.00	0.86	0.92	14
9	0.92	0.82	0.87	57
10	0.99	0.94	0.96	84
11	0.88	0.81	0.84	234
12	0.90	0.78	0.83	110
13	0.91	0.86	0.88	84
14	0.89	0.83	0.86	265
15	0.98	0.98	0.98	258
16	0.94	0.96	0.95	46
17	0.92	0.88	0.90	90
18	1.00	0.94	0.97	48
19	0.80	0.71	0.75	17
20	0.77	0.73	0.75	37
21	0.75	0.66	0.70	32
22	0.91	0.91	0.91	80
23	0.86	0.68	0.76	74
24	0.83	0.76	0.79	25
25	0.92	0.97	0.95	37
26	0.89	1.00	0.94	25
27	0.94	1.00	0.97	65
28	0.82	0.79	0.81	184
29	0.92	0.97	0.94	123
30	0.98	0.95	0.97	153
31	0.85	0.89	0.87	53
32	0.86	0.74	0.80	140
33	0.87	0.87	0.87	432
34	0.92	0.93	0.93	117
35	0.93	0.95	0.94	318
36	0.77	0.78	0.78	157
37	1.00	0.95	0.98	43
38	0.89	0.98	0.93	42

```
          39       0.70      0.38      0.49        37
          40       0.90      0.73      0.80        59
          41       0.75      0.78      0.76      1234
          42       0.87      0.63      0.73        71
          43       0.96      0.96      0.96       208
          44       0.65      0.42      0.51       231
          45       0.78      0.73      0.75       187
          46       0.85      0.83      0.84       548

   micro avg       0.86      0.84      0.85      7226
   macro avg       0.88      0.84      0.86      7226
weighted avg       0.86      0.84      0.85      7226
 samples avg       0.81      0.80      0.79      7226
```

```
/data/chhaag/conda/envs/myenv3/lib/python3.11/site-packages/sklearn/metrics/_classification.py:1565: UndefinedMetric
Warning: Precision is ill-defined and being set to 0.0 in samples with no predicted labels. Use `zero_division` para
meter to control this behavior.
  _warn_prf(average, modifier, f"{metric.capitalize()} is", len(result))
/data/chhaag/conda/envs/myenv3/lib/python3.11/site-packages/sklearn/metrics/_classification.py:1565: UndefinedMetric
Warning: Recall is ill-defined and being set to 0.0 in samples with no true labels. Use `zero_division` parameter to
control this behavior.
  _warn_prf(average, modifier, f"{metric.capitalize()} is", len(result))
/data/chhaag/conda/envs/myenv3/lib/python3.11/site-packages/sklearn/metrics/_classification.py:1565: UndefinedMetric
Warning: F-score is ill-defined and being set to 0.0 in samples with no true nor predicted labels. Use `zero_divisio
n` parameter to control this behavior.
  _warn_prf(average, modifier, f"{metric.capitalize()} is", len(result))
```

	precision	recall	f1-score	support
0	0.75	0.67	0.71	92
1	0.90	0.87	0.88	284
2	0.86	0.86	0.86	74
3	0.93	0.93	0.93	319
4	0.97	0.94	0.95	32
5	1.00	0.94	0.97	85
6	0.99	1.00	1.00	173
7	0.84	0.82	0.83	148
8	1.00	0.86	0.92	14
9	0.91	0.84	0.87	57
10	0.99	0.94	0.96	84
11	0.86	0.79	0.83	234
12	0.88	0.82	0.85	110
13	0.90	0.87	0.88	84
14	0.87	0.85	0.86	265
15	0.98	0.98	0.98	258
16	0.92	0.98	0.95	46
17	0.88	0.91	0.90	90
18	1.00	0.96	0.98	48
19	0.75	0.71	0.73	17
20	0.77	0.73	0.75	37
21	0.84	0.66	0.74	32
22	0.89	0.91	0.90	80
23	0.83	0.73	0.78	74
24	0.83	0.76	0.79	25
25	0.90	0.95	0.92	37
26	0.89	1.00	0.94	25
27	0.94	1.00	0.97	65
28	0.79	0.79	0.79	184
29	0.92	0.96	0.94	123
30	0.98	0.96	0.97	153
31	0.87	0.89	0.88	53
32	0.84	0.74	0.78	140
33	0.89	0.86	0.87	432
34	0.92	0.91	0.92	117
35	0.93	0.96	0.94	318
36	0.76	0.78	0.77	157
37	1.00	0.95	0.98	43
38	0.91	0.98	0.94	42

```
          39       0.79      0.41      0.54        37
          40       0.91      0.69      0.79        59
          41       0.74      0.81      0.77      1234
          42       0.82      0.69      0.75        71
          43       0.96      0.96      0.96       208
          44       0.60      0.48      0.53       231
          45       0.82      0.67      0.74       187
          46       0.86      0.82      0.84       548

   micro avg       0.86      0.84      0.85      7226
   macro avg       0.88      0.84      0.86      7226
weighted avg       0.86      0.84      0.85      7226
 samples avg       0.80      0.80      0.79      7226
```

```
/data/chhaag/conda/envs/myenv3/lib/python3.11/site-packages/sklearn/metrics/_classification.py:1565: UndefinedMetric
Warning: Precision is ill-defined and being set to 0.0 in samples with no predicted labels. Use `zero_division` para
meter to control this behavior.
  _warn_prf(average, modifier, f"{metric.capitalize()} is", len(result))
/data/chhaag/conda/envs/myenv3/lib/python3.11/site-packages/sklearn/metrics/_classification.py:1565: UndefinedMetric
Warning: Recall is ill-defined and being set to 0.0 in samples with no true labels. Use `zero_division` parameter to
control this behavior.
  _warn_prf(average, modifier, f"{metric.capitalize()} is", len(result))
/data/chhaag/conda/envs/myenv3/lib/python3.11/site-packages/sklearn/metrics/_classification.py:1565: UndefinedMetric
Warning: F-score is ill-defined and being set to 0.0 in samples with no true nor predicted labels. Use `zero_divisio
n` parameter to control this behavior.
  _warn_prf(average, modifier, f"{metric.capitalize()} is", len(result))
```

	precision	recall	f1-score	support
0	0.72	0.65	0.69	92
1	0.89	0.88	0.89	284
2	0.83	0.88	0.86	74
3	0.93	0.93	0.93	319
4	0.97	0.94	0.95	32
5	1.00	0.94	0.97	85
6	0.99	1.00	1.00	173
7	0.88	0.82	0.85	148
8	1.00	0.79	0.88	14
9	0.90	0.82	0.86	57
10	0.99	0.94	0.96	84
11	0.87	0.81	0.84	234
12	0.89	0.81	0.85	110
13	0.90	0.87	0.88	84
14	0.87	0.86	0.87	265
15	0.98	0.98	0.98	258
16	0.94	0.98	0.96	46
17	0.90	0.89	0.89	90
18	1.00	0.96	0.98	48
19	0.86	0.71	0.77	17
20	0.74	0.70	0.72	37
21	0.79	0.69	0.73	32
22	0.89	0.91	0.90	80
23	0.84	0.72	0.77	74
24	0.83	0.60	0.70	25
25	0.90	0.95	0.92	37
26	0.89	1.00	0.94	25
27	0.94	1.00	0.97	65
28	0.81	0.79	0.80	184
29	0.92	0.97	0.94	123
30	0.98	0.97	0.97	153
31	0.87	0.87	0.87	53
32	0.85	0.74	0.79	140
33	0.88	0.86	0.87	432
34	0.92	0.93	0.93	117
35	0.94	0.95	0.95	318
36	0.79	0.78	0.79	157
37	1.00	0.95	0.98	43
38	0.91	0.98	0.94	42

```
          39       0.75      0.41      0.53        37
          40       0.84      0.73      0.78        59
          41       0.76      0.76      0.76      1234
          42       0.88      0.69      0.77        71
          43       0.96      0.96      0.96       208
          44       0.60      0.42      0.50       231
          45       0.82      0.71      0.76       187
          46       0.87      0.81      0.84       548

   micro avg       0.87      0.83      0.85      7226
   macro avg       0.88      0.84      0.86      7226
weighted avg       0.86      0.83      0.85      7226
 samples avg       0.81      0.80      0.79      7226
```

```
/data/chhaag/conda/envs/myenv3/lib/python3.11/site-packages/sklearn/metrics/_classification.py:1565: UndefinedMetric
Warning: Precision is ill-defined and being set to 0.0 in samples with no predicted labels. Use `zero_division` para
meter to control this behavior.
  _warn_prf(average, modifier, f"{metric.capitalize()} is", len(result))
/data/chhaag/conda/envs/myenv3/lib/python3.11/site-packages/sklearn/metrics/_classification.py:1565: UndefinedMetric
Warning: Recall is ill-defined and being set to 0.0 in samples with no true labels. Use `zero_division` parameter to
control this behavior.
  _warn_prf(average, modifier, f"{metric.capitalize()} is", len(result))
/data/chhaag/conda/envs/myenv3/lib/python3.11/site-packages/sklearn/metrics/_classification.py:1565: UndefinedMetric
Warning: F-score is ill-defined and being set to 0.0 in samples with no true nor predicted labels. Use `zero_divisio
n` parameter to control this behavior.
  _warn_prf(average, modifier, f"{metric.capitalize()} is", len(result))
```

Out[11]:
```
TrainOutput(global_step=33890, training_loss=0.020655228110955295, metrics={'train_runtime': 5916.0644, 'train_sam
ples_per_second': 45.821, 'train_steps_per_second': 5.728, 'total_flos': 7.13529624692736e+16, 'train_loss': 0.020
655228110955295, 'epoch': 10.0})
```

In [12]:
```
# After training, the best model is automatically loaded and can be saved

model.save_pretrained("/data/chhaag/ICD_NLP/best_ICD_model")
```

In [13]:
```
import pandas as pd
import torch
from transformers import AutoTokenizer, AutoModelForSequenceClassification


# —— 1) Pick your model directory/name ————————————————————————————————
```

```
model_name = "/data/chhaag/ICD_NLP/best_ICD_model"


# ─── 2) Load tokenizer + model ───────────────────────────────────────────────
tokenizer = AutoTokenizer.from_pretrained(model_name)
model = AutoModelForSequenceClassification.from_pretrained(model_name)
model.eval()


# ─── 3) Send model to GPU if available ───────────────────────────────────────
device = torch.device("cuda" if torch.cuda.is_available() else "cpu")
model.to(device)  # now model parameters live on GPU (if cuda is available)

def predict_labels(text: str, threshold: float = 0.5) -> list[int]:
    """
    Tokenize 'text', move inputs to GPU (if using cuda), run model, return
    multi-label 0/1 list.
    """

    # 3a) Tokenize on CPU (the tokenizer itself returns CPU tensors)
    inputs = tokenizer(
        text,
        return_tensors="pt",
        padding=True,
        truncation=True,
        max_length=512,
    )
    # 3b) Move the tokenized inputs to the same device as the model:
    inputs = {k: v.to(device) for k, v in inputs.items()}

    # 3c) Run forward pass WITHOUT gradients
    with torch.no_grad():
        outputs = model(**inputs)

    # 3d) Extract logits (already on GPU if device="cuda")
    logits = outputs.logits[0]                 # shape: (num_labels,)
    probs = torch.sigmoid(logits)              # multi-label probabilities
    preds = (probs >= threshold).int()         # tensor of 0/1 on GPU or CPU
    return preds.cpu().tolist()                # move back to CPU as a Python list
```

```
# ── 4) Read your DataFrame ──────────────────────────────────────────────
df = pd.read_excel("/data/chhaag/ICD_NLP/data/icd_df_unseen_test_data.xlsx")


# ── 5) Apply predict_labels (each call now runs on GPU if available) ──────
df["predictions"] = df["text"].apply(predict_labels)


# ── 6) Expand prediction-lists into separate columns ────────────────────
existing_label_columns = ['A00_B99', 'C00_D48', 'D50_D90', 'E00_E90',
                          'E55', 'E65_E68', 'E78', 'F00_F99', 'F00_F03', 'F17',
                          'F30_F39', 'G00_G99', 'H00_H59', 'H60_H95', 'I00_I99',
                          'I10', 'I11_I14', 'I20_I25', 'I48', 'I50', 'I60_I69',
                          'I70', 'I83_I87', 'J00_J99', 'J00_J06', 'J30', 'J44',
                          'J45', 'K00_K93', 'K21_K30', 'K50_K64', 'K70_K87',
                          'L00_L99', 'M00_M99', 'M16_M19', 'M40_M54', 'N00_N99',
                          'N18', 'N40', 'Q00_Q99', 'S00_T98', 'ZR', 'T88_7_X49_Y57',
                          'verdacht', 'unklar', 'keine', 'Stn']
preds_df = pd.DataFrame(
    df["predictions"].tolist(),
    columns=existing_label_columns,
    index=df.index
)


# ── 7) Join predicted columns (with "_pred" suffix) to original DataFrame ──
df = df.join(preds_df.add_suffix("_pred"))


# ── 8) Save to Excel ─────────────────────────────────────────────
df.to_excel("/data/chhaag/ICD_NLP/data/icd_unseen_data_with_predictions.xlsx", index=False)
```

In [14]:

```
# EVALUATION

from sklearn.metrics import precision_recall_fscore_support, classification_report

# 1) Build arrays of ground truth and predictions:
pred_label_columns = [f"{c}_pred" for c in existing_label_columns]
y_true = df[existing_label_columns].values
y_pred = df[pred_label_columns].values
```

```
# 2) Per-label precision/recall/F1:
precision_per_label, recall_per_label, f1_per_label, support_per_label = \
    precision_recall_fscore_support(
        y_true, y_pred, average=None, zero_division=0
    )

print("Per-label metrics:")
for label, p, r, f, s in zip(
    existing_label_columns,
    precision_per_label,
    recall_per_label,
    f1_per_label,
    support_per_label
):
    print(f"{label:10s} | precision={p:.3f} | recall={r:.3f} | f1={f:.3f} | support={s}")

# 3) Macro / micro / weighted averages:
p_micro, r_micro, f1_micro, _ = precision_recall_fscore_support(
    y_true, y_pred, average="micro", zero_division=0
)
p_macro, r_macro, f1_macro, _ = precision_recall_fscore_support(
    y_true, y_pred, average="macro", zero_division=0
)
p_w, r_w, f1_w, _ = precision_recall_fscore_support(
    y_true, y_pred, average="weighted", zero_division=0
)

print("\nAggregated scores:")
print(f"Micro-avg    | precision={p_micro:.3f} | recall={r_micro:.3f} | f1={f1_micro:.3f}")
print(f"Macro-avg    | precision={p_macro:.3f} | recall={r_macro:.3f} | f1={f1_macro:.3f}")
print(f"Weighted-avg | precision={p_w:.3f}     | recall={r_w:.3f}     | f1={f1_w:.3f}")
```

```
Per-label metrics:
A00_B99    | precision=0.779 | recall=0.652 | f1=0.710 | support=92
C00_D48    | precision=0.895 | recall=0.926 | f1=0.910 | support=285
D50_D90    | precision=0.871 | recall=0.824 | f1=0.847 | support=74
E00_E90    | precision=0.944 | recall=0.903 | f1=0.923 | support=319
E55        | precision=1.000 | recall=1.000 | f1=1.000 | support=32
E65_E68    | precision=0.965 | recall=0.965 | f1=0.965 | support=85
E78        | precision=0.994 | recall=0.983 | f1=0.988 | support=173
F00_F99    | precision=0.922 | recall=0.797 | f1=0.855 | support=148
F00_F03    | precision=1.000 | recall=0.867 | f1=0.929 | support=15
F17        | precision=0.929 | recall=0.912 | f1=0.920 | support=57
F30_F39    | precision=0.964 | recall=0.952 | f1=0.958 | support=84
G00_G99    | precision=0.858 | recall=0.876 | f1=0.867 | support=234
H00_H59    | precision=0.903 | recall=0.845 | f1=0.873 | support=110
H60_H95    | precision=0.940 | recall=0.929 | f1=0.934 | support=84
I00_I99    | precision=0.861 | recall=0.842 | f1=0.851 | support=265
I10        | precision=0.969 | recall=0.981 | f1=0.975 | support=257
I11_I14    | precision=0.957 | recall=0.957 | f1=0.957 | support=46
I20_I25    | precision=0.895 | recall=0.944 | f1=0.919 | support=90
I48        | precision=0.957 | recall=0.917 | f1=0.936 | support=48
I50        | precision=0.643 | recall=0.529 | f1=0.581 | support=17
I60_I69    | precision=0.811 | recall=0.811 | f1=0.811 | support=37
I70        | precision=0.839 | recall=0.812 | f1=0.825 | support=32
I83_I87    | precision=0.905 | recall=0.950 | f1=0.927 | support=80
J00_J99    | precision=0.760 | recall=0.770 | f1=0.765 | support=74
J00_J06    | precision=0.700 | recall=0.583 | f1=0.636 | support=24
J30        | precision=0.865 | recall=0.865 | f1=0.865 | support=37
J44        | precision=0.917 | recall=0.880 | f1=0.898 | support=25
J45        | precision=0.955 | recall=0.969 | f1=0.962 | support=65
K00_K93    | precision=0.837 | recall=0.810 | f1=0.823 | support=184
K21_K30    | precision=0.944 | recall=0.959 | f1=0.952 | support=123
K50_K64    | precision=0.961 | recall=0.967 | f1=0.964 | support=153
K70_K87    | precision=0.981 | recall=0.962 | f1=0.971 | support=53
L00_L99    | precision=0.847 | recall=0.823 | f1=0.835 | support=141
M00_M99    | precision=0.843 | recall=0.843 | f1=0.843 | support=433
M16_M19    | precision=0.956 | recall=0.932 | f1=0.944 | support=117
M40_M54    | precision=0.930 | recall=0.921 | f1=0.926 | support=318
N00_N99    | precision=0.808 | recall=0.803 | f1=0.805 | support=157
N18        | precision=0.952 | recall=0.909 | f1=0.930 | support=44
N40        | precision=0.933 | recall=1.000 | f1=0.966 | support=42
Q00_Q99    | precision=0.714 | recall=0.405 | f1=0.517 | support=37
```

```
S00_T98     | precision=0.780 | recall=0.533 | f1=0.634 | support=60
ZR          | precision=0.727 | recall=0.744 | f1=0.735 | support=1234
T88_7_X49_Y57 | precision=0.860 | recall=0.690 | f1=0.766 | support=71
verdacht    | precision=0.922 | recall=0.962 | f1=0.941 | support=208
unklar      | precision=0.617 | recall=0.481 | f1=0.540 | support=231
keine       | precision=0.771 | recall=0.647 | f1=0.703 | support=187
Stn         | precision=0.847 | recall=0.850 | f1=0.849 | support=548

Aggregated scores:
Micro-avg    | precision=0.854 | recall=0.836 | f1=0.845
Macro-avg    | precision=0.877 | recall=0.840 | f1=0.856
Weighted-avg | precision=0.852    | recall=0.836    | f1=0.843
```

In [ ]:
